# Estrogen Receptor β Agonists Differentially Affect the Growth of Human Melanoma Cell Lines

**DOI:** 10.1371/journal.pone.0134396

**Published:** 2015-07-30

**Authors:** Monica Marzagalli, Lavinia Casati, Roberta M. Moretti, Marina Montagnani Marelli, Patrizia Limonta

**Affiliations:** 1 Department of Pharmacological and Biomolecular Sciences, Università degli Studi di Milano, Milano, Italy; 2 Department of Medical Biotechnologies and Translational Medicine, Università degli Studi di Milano, Milano, Italy; University of Salerno, Faculty of Medicine and Surgery, ITALY

## Abstract

**Background:**

Cutaneous melanoma is an aggressive malignancy; its incidence is increasing worldwide and its prognosis remains poor. Clinical observations indicate that estrogen receptor β (ERβ) is expressed in melanoma tissues and its expression decreases with tumor progression, suggesting its tumor suppressive function. These experiments were performed to investigate the effects of ERβ activation on melanoma cell growth.

**Methods and Results:**

Protein expression was analyzed by Western blot and immunofluorescence assays. Cell proliferation was assessed by counting the cells by hemocytometer. ERβ transcriptional activity was evaluated by gene reporter assay. Global DNA methylation was analyzed by restriction enzyme assay and ERβ isoforms were identified by qRT-PCR. We demonstrated that ERβ is expressed in a panel of human melanoma cell lines (BLM, WM115, A375, WM1552). In BLM (NRAS-mutant) cells, ERβ agonists significantly and specifically inhibited cell proliferation. ERβ activation triggered its cytoplasmic-to-nuclear translocation and transcriptional activity. Moreover, the antiproliferative activity of ERβ agonists was associated with an altered expression of G1-S transition-related proteins. In these cells, global DNA was found to be hypomethylated when compared to normal melanocytes; this DNA hypomethylation status was reverted by ERβ activation. ERβ agonists also decreased the proliferation of WM115 (BRAF V600D-mutant) cells, while they failed to reduce the growth of A375 and WM1552 (BRAF V600E-mutant) cells. Finally, we could observe that ERβ isoforms are expressed at different levels in the various cell lines. Specific oncogenic mutations or differential expression of receptor isoforms might be responsible for the different responses of cell lines to ERβ agonists.

**Conclusions:**

Our results demonstrate that ERβ is expressed in melanoma cell lines and that ERβ agonists differentially regulate the proliferation of these cells. These data confirm the notion that melanoma is a heterogeneous tumor and that genetic profiling is mandatory for the development of effective personalized therapeutic approaches for melanoma patients.

## Introduction

The incidence of cutaneous melanoma is increasing worldwide [1] and its prognosis is still poor [2]. Cytotoxic drugs, dacarbazine or temozolomide, were reported to be associated with serious side effects and with development of resistance. Interleukin-2 or interferon-α yielded limited response rates with no benefit on overall survival or progression-free survival [3]. Patients treated with either mutated BRAF or MEK inhibitors, despite initial excellent response rates, showed a rapid relapse [4]. The anti-CTLA-4 (cytotoxic T-lymphocyte antigen 4) monoclonal antibody ipilimumab, despite its effectiveness, has side effects that can be non-reversible (autoimmune responses, bowel perforation) [5]. Thus, the elucidation of the molecular mechanisms of melanoma growth and progression is urgently needed for the identification of novel targets of intervention for the prevention and therapy of this disease [6].

The association of estrogens with tumor development has been investigated for many years. Estrogens exert their effects through the binding to two estrogen receptor (ER) subtypes, ERα and ERβ. These receptors are structurally similar, however they differ in the ligand binding domain and this confers them selectivity for different ligands [7]. After being activated by the binding of 17β-estradiol (E_2_) or of synthetic compounds these receptors exert their effects at the nuclear level through the binding to estrogen response elements on DNA to regulate the expression of specific target genes [7,8].

Both ER subtypes are expressed in different cells/tissues where they are involved in the control of specific physiological functions [9]. In addition, the activation of the two receptor subtypes elicits opposite effects on cancer growth and progression. ERα is associated with a proliferative activity while ERβ exerts a significant antitumor effect, being considered a protein with tumor suppressive functions [7,10,11]. These observations indicate that the actions of estrogens on cancer growth might depend on the relative ERα/ERβ ratio in a given tumor cell/tissue [12]. The expression of ERβ was found to be reduced in several cancer cells [13,14]. Moreover, overexpression of ERβ or its activation by means of agonistic ligands were reported to inhibit cell proliferation in different tumor cells, both classically related (breast, ovarian, and prostate cancer) [15–17] and unrelated (colon cancer, mesothelioma, cholangiocarcinoma, lymphoma) [18–21] to the reproductive system. Research is now focusing on the development and evaluation of selective ERβ ligands that might increase the activity of this receptor in tumors [8]. The expression of the different variants of this receptor (ERβ1, corresponding to ERβ, ERβ2 and ERβ5) and their specific role in tumor growth are also under investigation [22].

Increasing evidence suggests that ERβ might play a fundamental role also in the development and progression of melanoma [23]. Population data have established that women have survival advantage over men [24,25]. Moreover, men were reported to express lower levels of ERβ than women in both melanoma and healthy tissues [26]. More importantly, the expression levels of ERβ inversely correlate with melanoma progression [26,27]. These observations strongly support an antitumor activity of ERβ also in melanoma.

Alterations of DNA methylation, histone modifications, and modified expression of microRNAs are well-established epigenetic mechanisms of cell neoplastic transformation. In particular, melanoma cells present aberrant DNA methylation patterns with DNA hypermethylation at the level of CpG islands in the promoter of tumor suppressor genes (leading to their inactivation) and global DNA hypomethylation (contributing to genomic instability). Hypomethylation of specific genes was also shown, leading to the overexpression of normally silenced oncogenes [28,29]. Global DNA hypomethylation was reported to correlate with melanoma progression to the most aggressive phase and with less favourable clinical outcomes [28,30]. Epigenetic modifications and their reversibility by means of pharmacologic interventions might offer promising novel therapies for melanoma patients.

In this paper, we demonstrate that ERβ (but not ERα) is expressed in a panel of human melanoma cell lines (BLM, WM115, A375, WM1552). In BLM (NRAS-mutant) cells, activation of this receptor, by means of agonistic ligands, induces its translocation into the nucleus and initiation of transcriptional activity, thereby significantly and specifically decreasing melanoma cell proliferation. This antitumor activity is accompanied by an altered expression of the proteins involved in the G1-S progression of the cell cycle, but not by triggering of the apoptosis pathway. Moreover, in these cells, ERβ agonists increase global DNA methylation, reverting the observed DNA hypomethylation status of melanoma cells compared to normal melanocytes.

ERβ agonists also exert a tumor suppressor activity in WM115 (BRAF V600D-mutant) cells, while they fail to reduce cell proliferation in A375 and WM1552 (BRAF V600E-mutant) cells. Moreover, ERβ isoforms show different levels of expression in the various cell lines. The possible reasons for the differential effects of ERβ agonists on the growth of the melanoma cells (oncogenic mutations, receptor isoform expression) are discussed.

## Materials and Methods

### Cell culture and reagents

The human BLM (NRAS-mutant, BRAF-wild type) melanoma cell line was provided by Dr. G.N. van Muijen (Department of Pathology, Radbound University Nijmegen Medical Center, Nijmegen, The Netherlands). This cell line is a subline of BRO melanoma cells isolated from lung metastases after subcutaneous inoculation of nude mice with BRO cells [31]. This cell line was previously utilized in the authors' laboratory to study the antitumor activity of gonadotropin-releasing hormone receptors in melanoma cells [32,33]. The human WM115 (BRAF V600D-mutant) and WM1552 (BRAF V600E-mutant) melanoma cell lines, kindly provided by Dr. R. Giavazzi (Department of Oncology, Mario Negri Institute for Pharmacological Research, Milano, Italy) were originally from Dr. M. Herlyn (Wistar Institute, Philadelphia, PA) [34,35]. The human IGR-39 (BRAF V600E-mutant) melanoma cell line, kindly provided by Dr. C.A. La Porta, was originally from Leibniz-Institut DSMZ-Deutsche Sammlung von Mikroorganismen and Zellkulturen GmbH (38124 Braunschweig, Germany). The human MCF-7 breast cancer, A375 (BRAF V600E-mutant) and embryonic kidney (HEK) 293 cells were from American Type Culture Collection (ATCC, Manassas, VA, USA). Primary human melanocytes were provided by Dr. F. Crovato (Regional Reference Centre for Human Epidermis *in vitro* Culture and Bank for Tissue Crypreservation, Niguarda Hospital, Milano, Italy). Stocks of cells were stored frozen in liquid nitrogen and kept in culture for no more than 10–12 weeks.

MCF-7 and WM115 cells were routinely grown in RPMI-1640 medium supplemented with 10% FBS, glutamine (1 mmol/l) and antibiotics (100 IU/ml, penicillin G sodium and 100 μg/ml streptomycin sulfate). BLM, A375, WM1552, IGR-39 and HEK293 cells were routinely cultured in DMEM medium supplemented with 10% FBS, glutamine and antibiotics, as described above. Cells were cultured in humidified atmosphere of 5% CO_2_/95% air at 37°C.

17β-Estradiol was purchased from Sigma-Aldrich (Milano, Italy); the ER antagonist ICI 182,780 and the ERβ agonist DPN (diarylpropionitrile) were from Tocris Biosciences (Bristol, UK). The selective ERβ agonists KB1, KB2, and KB4 were kindly provided by Dr. S. Nilsson (Karo Bio AB, Novum, SE-141 57 Huddinge, Sweden). KB1 (also known as KB9520) was previously utilized in different studies as the specific ligand of ERβ to investigate the antitumor activity of the receptor in cancer cells [14,20,21,36]. The selective activity of this class of compounds as ERβ ligands was previously demonstrated [37]. The compounds can be obtained following contact with Karo Bio AB and after signing of a Material Transfer Agreement together with a detailed protocol of planned study. A fee covering the cost of compound synthesis will be charged.

### ERβ overexpression

The plasmid pCMV5-hERbeta, expressing human wild type ERβ, was kindly provided by Dr. A. Maggi (Department of Pharmacological and Biomolecular Sciences, University of Milano, Milano, Italy). BLM cells were plated (8 x10^4^ cells/dish) in 6-well plates in DMEM complete medium. After 48 h, the medium was replaced with DMEM medium and the cells were transiently transfected using Lipofectamine 2000 (Invitrogen, Monza, Italy), according to the manufacturer’s protocol. After 24–72 h of transfection the cells were lysed in RIPA buffer for protein extraction.

### Western blot analysis

Cells were lysed in RIPA buffer (0.05 M Tris-HCl pH 7.7, 0.15 M NaCl, 0.8% SDS, 10 mM EDTA, 100 μM NaVO_4_, 50 mmol/L NaF, 0.3 mM PMSF, 5 mM iodoacetic acid) containing leupeptin (50 μg/ml), aprotinin (5μl/ml) and pepstatin (50 μg/ml). Protein concentration in lysates was determined using the BCA method. Protein extracts (20–30 μg) were resuspended in Sample buffer (0.5 M Tris.HCl pH 6.8, 20% glycerol, 10% SDS, 0.2% 2β-mercaptoethanol, 0.05% blue bromophenol) and heated at 95°C for 5 min. Following electrophoretic separation by 10% SDS-PAGE, proteins were transferred onto nitrocellulose membranes. Membranes were blocked in nonfat dry milk (7.5% for ERβ and 5% for ERα) prior to incubation at 4°C overnight with the primary antibodies (1:1000): rabbit polyclonal antibody SC 8974 (clone H-150, Santa Cruz Biotechnology, Santa Cruz, CA) and mouse monoclonal antibody ab288 (clone 14C8, Abcam, Cambridge, MA) for ERβ; rat monoclonal antibody Ab-21 (clone H222, Thermo Scientific, Waltham, MA) for ERα. Detection was done using a horseradish-peroxidase-conjugated secondary antibody and enhanced chemiluminescence reagents (GE Healthcare, Life Sciences, Milano, Italy).

To investigate the effects of ERβ activation on the expression of cell cycle/apoptosis-related proteins, BLM cells were plated (5x10^5^ cells/dish) in 10-cm dishes, in standard culture conditions, for 48 h. Medium was then changed to phenol red free medium supplemented with 10% charcoal stripped FBS and treated with DPN (10^−8^ M) for 24, 48, or 72 h. Protein preparations were then processed for Western blotting, as described above, with the following primary antibodies: mouse monoclonal antibody against cyclin D1 (1:2000; clone DCS-6), mouse monoclonal antibody against cyclin D3 (1:1000; clone DCS22), rabbit monoclonal antibody against p21^Waf1/Cip1^ (1:1000; clone 12D1), rabbit monoclonal antibody against p27^Kip1^ (1:1000; clone D69C12), rabbit monoclonal antibody against CDK4 (1:1000; clone D9G3E), mouse monoclonal antibody against CDK6 (1:2000, clone DCS83), rabbit monoclonal antibody against caspase-3 (1:500, clone 8G10), and rabbit monoclonal antibody against cleaved caspase-3 (Asp175; 1:500, clone 5A1E). All these primary antibodies were from Cell Signaling Technology (Danvers, MA). Detection was done using a horseradish peroxidase-conjugated anti-mouse or a horseradish peroxidase-conjugated anti-rabbit secondary antibody (Santa Cruz Biotechnology), according to the primary antibody used, and enhanced chemiluminescence ECL-Prime reagents (GE Healthcare, Life Sciences). In each experiment, actin expression was evaluated as a loading control, using the goat polyclonal anti-human antibody (1:1000; I-19, sc-1616, Santz Cruz Biotechnology) as the primary antibody. Detection was done using a horseradish peroxidase-conjugated anti-goat secondary antibody (Santa Cruz Biotechnology) and enhanced chemiluminescence reagents, as described above. The experiments were repeated three times.

### Cell proliferation assays

BLM melanoma cells were plated (15x10^3^ cells/dish) in 6-cm dishes in DMEM complete medium. After 48 h, the medium was replaced with phenol red free medium supplemented with 10% charcoal stripped FBS. Cells were then treated as follows: i) DPN (10^−9^, 5x10^-9^, 10^−8^, 5x10^-8^, 10^−7^ M); E_2_ (10^−9^,10^−8^, 10^−7^ M); KB1, KB2, or KB4 (10^−9^, 10^−8^, 10^−7^ M); KB1 (10^−9^, 5x10^-9^, 10^−8^, 5x10^-8^, 10^−7^ M) every 48 h, for three times; ii) ICI 182,780 (10^−6^ M) for 1 h, followed by DPN, E_2_, or K1 (10^−8^ M) every 48 h, for three times. Cells were then harvested and counted by hemocytometer.

Experiments were also performed on IGR-39 (expressing almost undetectable levels of ERβ) and on A375, WM1552 and WM115 melanoma cells. IGR-39, A375 and WM1552 cells were treated with DPN (10^−9^, 10^−8^, 10^−7^ M); WM115 cells were treated with DPN, E_2_ or KB1 (10^−10^, 10^−9^, 10^−8^, 10^−7^ M) as described above. Each proliferation assay was repeated three-five times.

### Immunofluorescence assay

BLM cells (2x10^4^ cells) were seeded on 13-mm diameter coverslips in DMEM complete medium. After 48 h, the medium was replaced with phenol red free medium supplemented with 10% charcoal stripped FBS. Cells were then treated with either DPN or E_2_ (10^−8^ M) for 24 h, fixed with 4% paraformaldehyde in 2% sucrose-PBS for 15 min and permeabilized with 0.2% PBS/Triton buffer (1 mM PBS, 300 mM sucrose, 50 mM NaCl, 3 mM MgCl_2_, 0.5% Triton X-100) for 20 min at room temperature and stained with the primary rabbit anti-human ERβ polyclonal antibody SC 8974 (1:50; clone H-150, Santa Cruz Biotechnology), followed by FITC-conjugated goat anti-rabbit secondary antibody Alexa Fluor 488 (1:2000; Molecular Probes Inc., Eugene, OR). Labeled cells were examined under a Zeiss Axiovert 200 microscope with a 63x/1.4 objective lens linked to a Coolsnap Es CCD camera (Roper Scientific-Crisel Instruments, Roma, Italy). The experiment was repeated three times.

### ERβ transcriptional activity assay

BLM cells, seeded in 24-well plates (5x10^4^ cells/well) in phenol red free DMEM medium supplemented with 10% charcoal stripped FBS, were transfected using Lipofectamine 2000 (Life Technologies, Monza, Italy), according to the manufacturer’s protocol [38]. The following constructs were cotransfected: pVERE-tk-Luc (1μg), the reporter plasmid encoding the firefly luciferase reporter gene under the control of the estrogen response element (ERE; kindly provided by Dr. A. Maggi, Department of Pharmacological and Biomolecular Sciences, University of Milano, Milano, Italy), to evaluate the transcriptional activity of ERβ, and pCMVβ (0.4 μg), the reporter plasmid encoding the β-galactosidase (Clontech, Jesi, Italy), as the internal control plasmid. Efficiency of transfection was evaluated by fluorescent microscopy by transfecting the plasmid vector pCMV-pEGFP-N1 (Clontech).

ERβ transcriptional activity was measured using the neolite system (PerkinElmer, Boston, MA), according to the manufacturer’s protocols. Briefly, 6 h after transfection, BLM cells were treated with DPN (10^−8^ M), E_2_ (10^−8^ M), or ethanol (controls) for 24 h. Cell medium was removed and 175 μl of fresh medium were added to each well. Neolite luciferase reagent (175 μl) was added to each sample and firefly luminescence was read after 10 min. At the end of this step, 100 μl of the lysate were added in a microcentrifuge tube containing the assay buffer with ortho-Nitrophenyl-β-galactoside (ONPG). If β-galactosidase is present, it hydrolyzes the ONPG molecule into galactose and ortho-nitrophenol (yellow color). The samples were transferred into a 96-well plate and analyzed in an EnSpire Multimode Plate reader (PerkinElmer) at 420 nm. Data are expressed as the mean of the ratio (± SE) between luminescence of the experimental reporter (firefly luciferase activity) and that of the control reporter (β-galactosidase activity) and are the mean values from three replicates of three distinct experiments.

### Methylation analysis of GC-rich regions

DNA from human melanocytes and BLM cells was extracted using Qiagen column methods (Qiagen, Milano, Italy) according to the manufacturer's protocol. DNA quality and concentration was evaluated by measuring the 260/280 nm optical ratio using Nanodrop 2000 (OD260/280).

Digestion of genomic DNA with restriction enzymes RsaI, MspI and HpaII (Euroclone, Pero, Milano, Italy) was performed, as previously described [39]. For each DNA sample, 2 restriction digests were performed: one with RsaI and MspI, and one with RsaI and HpaII. RsaI is methylation insensitive, while MspI and HpaII are sensitive to DNA methylation and are able to cut only unmethylated restriction sites. In general, MspI will not cut if the external cytosine is methylated, and HpaII will not cut if any of the two cytosines is methylated [39,40]. Global DNA digestion was performed o/n at 37°C. Restriction digests were performed with 1 μg of DNA and 5 units of RsaI in Roche buffer L. After 1 h incubation at 37°C, 2.5 unit aliquots of MspI or HpaII were added, 2 h apart. Total incubation time was 18 h. The enzymes were inactivated by 10-min incubation at 65°C, and the digests were used for PCR using a single primer (5′-AACCCTCACCCTAACCCCGG-3′) that arbitrarily binds within GC-rich regions of DNA. Samples were resolved on 1% agarose gel. The intensity of the band was determined using ImageLab (Chemidoc Imager, Bio-Rad, Segrate, Milano, Italy). Data are expressed as the MspI/RsaI or HpaII/RsaI ratios relative to the intensity of the bands.

To investigate the effects of ERβ activation on the global DNA methylation profile of melanoma cells, BLM cells, seeded in 6-well plates (3x10^4^ cell/well), in phenol red free DMEM medium supplemented with 10% charcoal stripped FBS, were treated with DPN (10^−8^M), E_2_ (10^−8^M), or ethanol (controls) for 24 or 48 h. The analysis of DNA methylation profile was performed as described above. Each experiment was repeated three times.

### ERβ isoform expression by quantitative PCR

Total RNA from BLM, A375 and WM115 cells was isolated by RNeasy MINI kit (Qiagen) for the evaluation of the expression of ERβ isoforms; RNA pellets were dissolved in sterile distilled water and their concentrations were assessed using Nanodrop 2000 (OD260/280). Specific set of primers for each ERβ isoform were synthesized (Sigma-Aldrich) and utilized, as previously described by Collins and coworkers [41]. Real time-DNA amplification for ERβ was performed in CFX96 Bio-Rad using 20 μl of total volume. The efficiency of each set of primer was evaluated in preliminary experiment and it was found close to 100% for target genes and for the housekeeping gene glyceraldehyde-3-phosphate dehydrogenase (GAPDH). 600 ng of total RNA were retrotranscribed using the IScript Supermix kit (Bio-Rad), according to the manufacturer’s protocol. The reaction was carried out on 40 ng of total cDNA using SYBR chemistry (iTAQ Universal SYBR green supermix, Bio-Rad) according to the manufacturer’s protocol. Real-time PCR was run according to the following protocol: an initial step of 30 sec at 95°C followed by 40 cycles of 5 sec at 95°C and 30 sec at 60°C. A dissociation stage with a melt curve analysis was also performed. Three replicates were performed for each experimental point and experiments were repeated three times. Gene expression was quantified using the comparative threshold-cycle (Ct) method considering that the targets and the reference genes have the same amplification efficiency (near to 100%) [42]. In each cell line, ΔCt values (difference between target and reference gene Ct) for ERβ2 or ERβ5 were compared with ERβ1 ΔCt value in the same cell line.

### Statistical analysis

When appropriate, data were analyzed by Dunnet's or Bonferroni's test, after one-way analysis of variance. A *P* value <0.05 was considered statistically significant.

## Results

### Expression of ERβ in human melanoma cell lines

**Fig 1:**
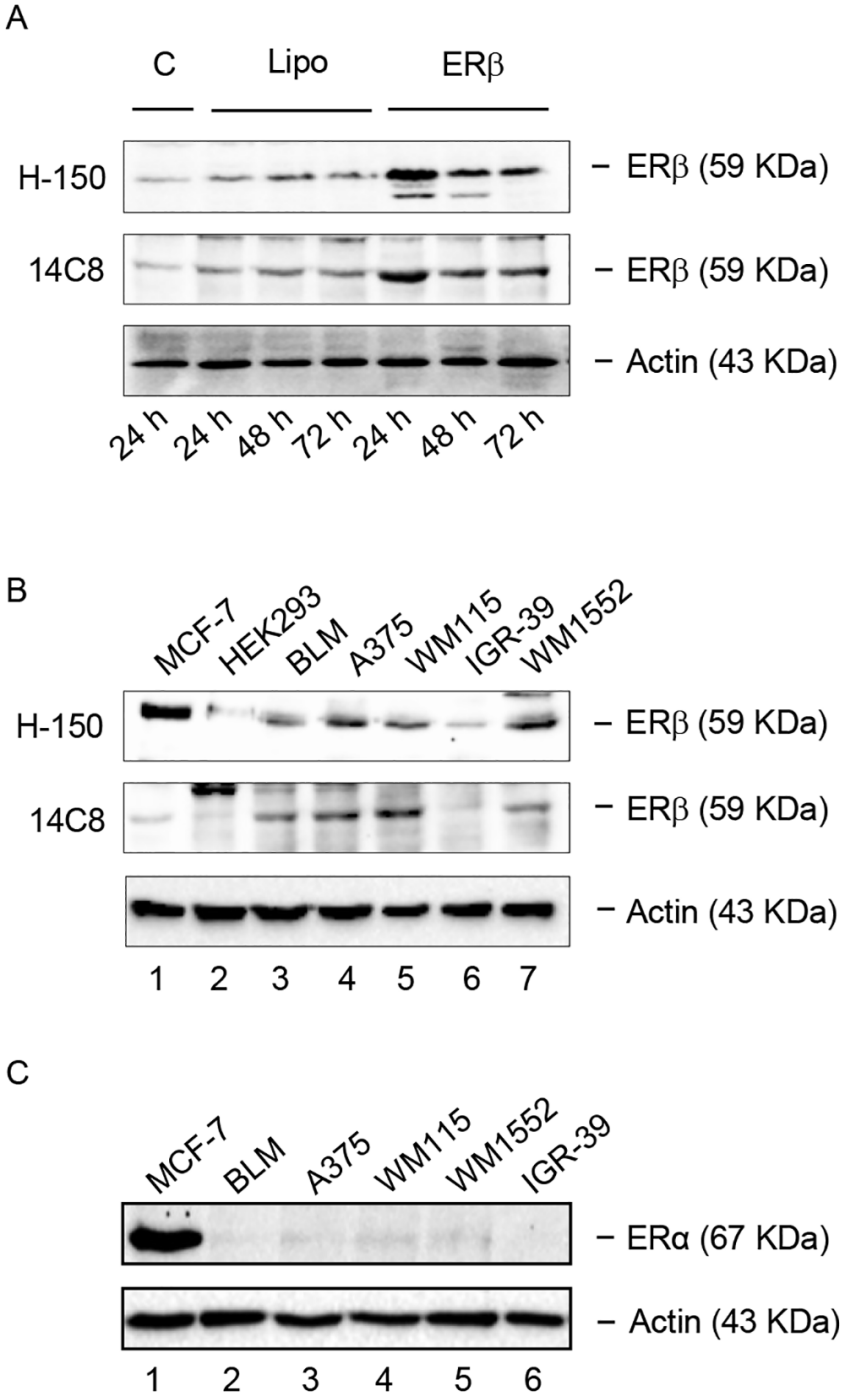
ERβ, but not ERα, is expressed in human melanoma cells. **(A)** As a positive control, the expression of ERβ was evaluated by Western blot analysis in human BLM melanoma cells engineered to overexpress the receptor subtype protein, utilizing two primary antibodies: H-150 (Santa Cruz) and 14C8 (Abcam). A band corresponding to the receptor protein (59 kDa) was detected in basal conditions, both in control (C) and in Lipofectamine (Lipo) treated BLM cells. As expected, the intensity of this band was found to be significantly increased after ERβ overexpression (24–72 h), with the highest level of expression at 24 h. **(B)** By Western blot analysis, utilizing the two primary antibodies H-150 and 14C8, ERβ was found to be expressed at high levels in human BLM, A375, WM115, WM1552 melanoma cell lines (lanes 3, 4, 5, 7), while the human IGR-39 melanoma cell line expressed the receptor at almost undetectable levels (lane 6). ERβ was also expressed in human MCF-7 breast cancer cells, utilized as a positive control (lane 1), but it was not expressed in the human HEK293, utilized as a negative control. **(C)** On the other hand, all the human melanoma cells lines tested (lanes 2–6) did not express the ERα receptor isoform, which was expressed only in the control cell line (MCF-7, lane 1). β-actin was utilized as a loading control. For each analysis, one representative of three different experiments, which gave similar results, is shown.

The expression of ERβ in human melanoma cell lines was analyzed by Western blot assay utilizing two primary antibodies: H-150 (Santa Cruz Biotechnology) and 14C8 (Abcam). First, in order to obtain an appropriate positive control, ERβ was evaluated in BLM melanoma cells engineered to overexpress the receptor protein. Fig 1A shows that a protein band, corresponding to the molecular weight of 59 kDa, is expressed in BLM cells either in normal culture conditions (C) or in the presence of Lipofectamine (Lipo), at 24–72 h. More importantly, Fig 1A also shows that the expression levels of this protein band are sharply increased in BLM cells overexpressing the receptor (ERβ) at 24 after transfection, and slightly decrease at 48 and 72 h. These data, obtained with the two different antibodies, confirm that the molecular weight of this receptor subtype corresponds to 59 kDa, as previously reported [43,44].

ERβ expression was then analyzed, utilizing the two primary ERβ antibodies (H-150 and 14C8) in a panel of human melanoma cell lines. Fig 1B shows that a specific band corresponding to the molecular weight of 59 kDa, is expressed in BLM (lane 3; confirming the data reported in Fig 1A), A375 (lane 4), WM115 (lane 5) and WM1552 (lane 7) human melanoma cells. Fig 1B also shows that in the human IGR-39 melanoma cell line (lane 6) the receptor is expressed at almost undetectable levels. The molecular weight of the protein band detected in the melanoma cell lines corresponds to that found in human MCF-7 breast cancer cells (positive control; lane 1). As expected, no band of this size could be detected in the HEK293 cells (negative control; lane 2), confirming previous observations [45]. It should be underlined that, when evaluated with the 14C8 primary antibody, the level of expression of the receptor in MCF-7 cells was found to be low, and this agrees with previous data in the literature [46,47]. On the other hand, in these cells the receptor seems to be expressed at higher levels when evaluated with the H-150 antibody. At present, the reason for this discrepancy is unclear; however, it might be due to a different degree of specificity of the two antibodies.

No band corresponding to ERα (67 kDa) could be detected in any of the melanoma cell lines analyzed (Fig 1C, lanes 2–6), confirming previous observations [48]; as expected, this estrogen receptor subtype was expressed at high levels in human MCF-7 breast cancer cells (positive control; Fig 1, lane 1). These data indicate that ERβ, but not ERα, is the estrogen receptor subtype expressed in most human melanoma cells.

### ERβ agonists inhibit the proliferation of BLM melanoma cells

**Fig 2:**
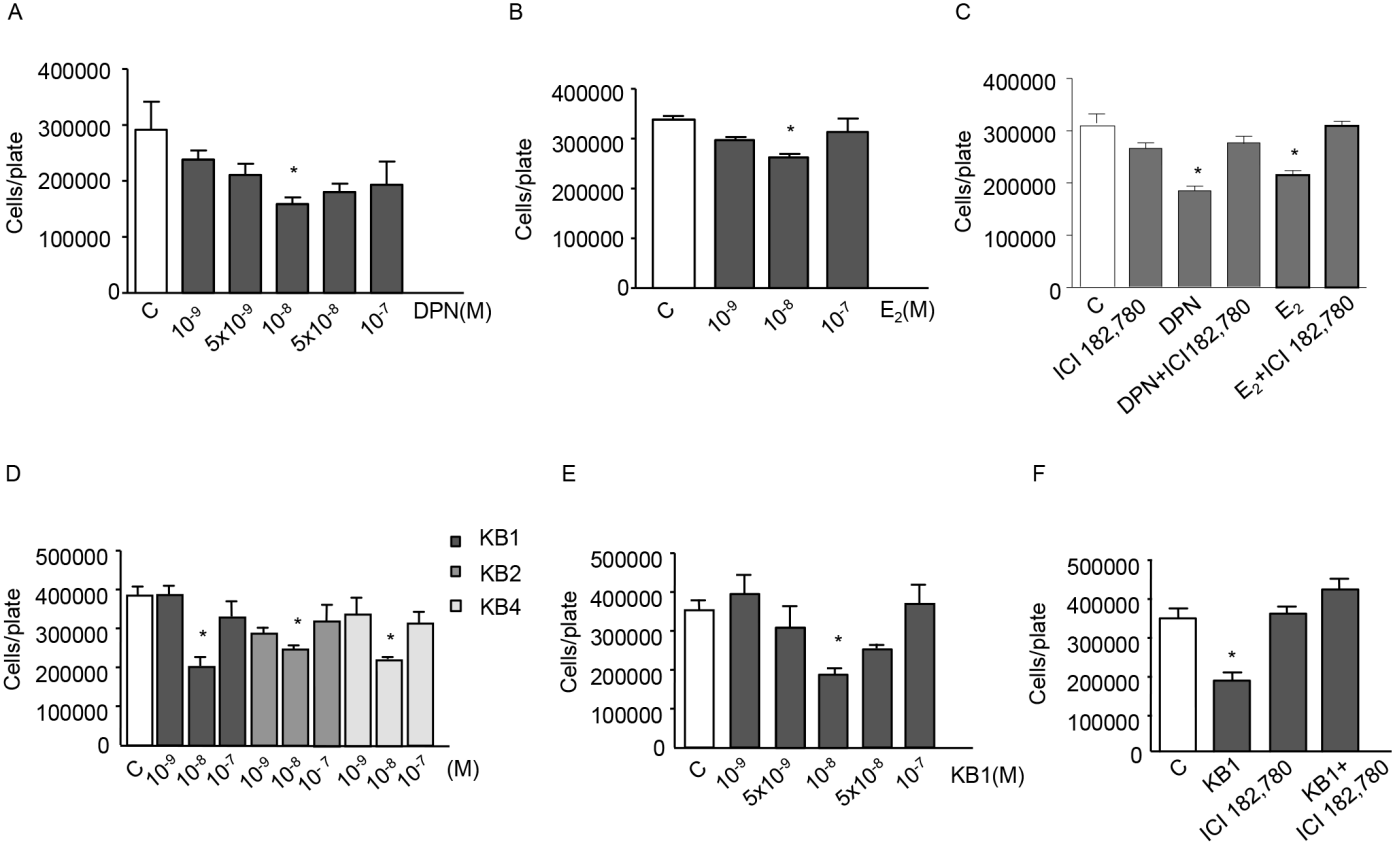
ERβ agonists significantly and specifically inhibit the proliferation of BLM melanoma cells. **(A)** BLM cells were treated with different doses of the classical ERβ agonist DPN every 48 h for three times. DPN significantly decreased cell proliferation at the dose of 10^−8^ M. **(B)** Similar results were obtained when the cells were treated with E_2_. **(C)** The antiproliferative effect of both ERβ ligands (10^−8^ M) was found to be specific since it was completely abrogated by cotreatment of the cells with the ER antagonist ICI 182,780 (10^−6^ M). **(D)** BLM cells were treated with KB1, KB2, or KB4 (10^−9^, 10^−8^, 10^−7^ M) every 48 h for three times. All three ERβ ligands significantly reduced cell proliferation at the dose of 10^−8^ M. **(E)** BLM cells were treated with KB1, at different doses (10^9^−10^−7^ M). The ERβ agonists decreased cell growth, being significantly effective at the dose of 10^−8^ M. **(F)** The antiproliferative activity of KB1 (10^−8^ M) was found to be specific since it was completely abrogated by cotreatment of the cells with the ER antagonist ICI 182,780. Each experimental group consisted of six replicates and each experiment was repeated three-five times. Results are given as cell number/plate. Data represent mean values ± SEM. *P<0.05. C, controls.

Experiments were first performed to investigate the effects of ERβ activation on the growth of BLM melanoma cells, expressing ERβ. The selective ERβ agonist DPN decreased BLM cell proliferation at the concentrations of 10^−9^ and 5x10^-9^ M, being significantly effective at the dose of 10^−8^ M (Fig 2A). This significant effect was followed by a decline at concentrations of 5x10^-8^ and 10^−7^ M. Accordingly, the natural estrogenic ligand E_2_ exerted a significant antiproliferative effect on BLM cell proliferation at the concentration of 10^−8^ M (Fig 2B). The antiproliferative activity of both DPN and E_2_ (10^−8^ M) was completely counteracted by cotreatment of the cells with the ER antagonist ICI 182,780 (10^−6^ M) (Fig 2C).

Experiments were also performed with different ERβ agonists (KB1, KB2, KB4). We found that all these compounds significantly inhibit BLM cell proliferation at the concentration of 10^−8^ M (Fig 2D); the dose-response curve obtained after treating the cells with different concentrations of KB1 was similar to that obtained with DPN (Fig 2E *vs*. Fig 2A). This effect was completely counteracted by the ER antagonist ICI 182,780 (10^−6^ M) (Fig 2F).

These data demonstrate that ERβ activation is associated with antiproliferative activity in BLM melanoma cells, with 10^−8^ M being the most effective dose, as previously reported for different tumor cells [14,18,20,21,49–51]. A curve of the dose-response effect of ERβ agonists on cancer cell proliferation, similar to that here shown, has been previously reported for cholangiocarcinoma and mesothelioma cells [14,20]. On the basis of these results, the concentration of 10^−8^ M was selected for the subsequent studies.

### Activation of ERβ induces its cytoplasmic-to-nuclear translocation and transcriptional activity in BLM cells

**Fig 3:**
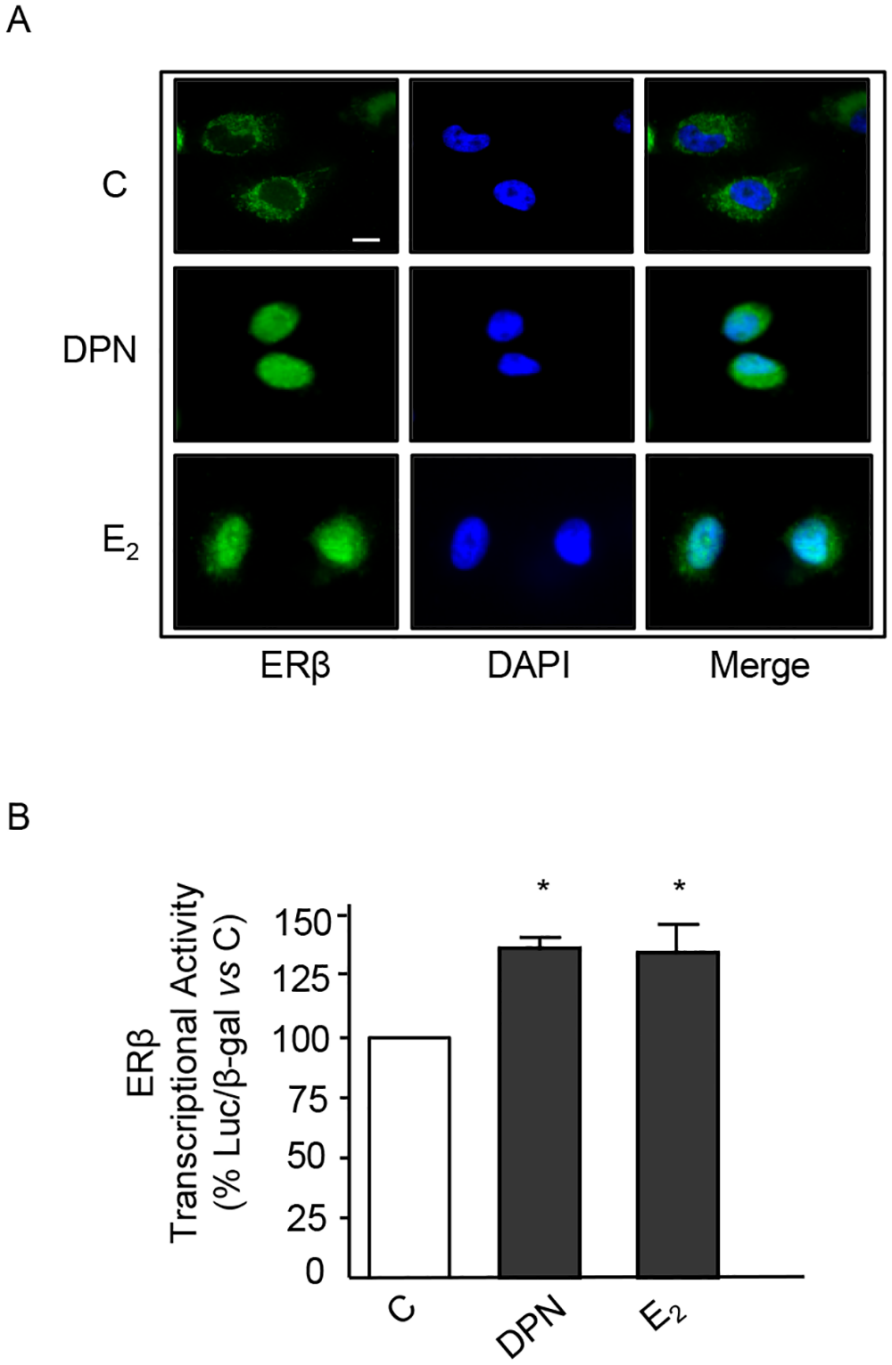
ERβ ligands trigger cytoplasmic-to-nuclear translocation of ERβ and induce its transcriptional acitivity in BLM melanoma cells. **(A)** Immunofluorescence assay of ERβ intracellular localization. In control BLM melanoma cells, ERβ is mainly localized at the cytoplasmic level. Treatment of the cells with either DPN or E_2_ (10^−8^ M, for 24 h) induces ERβ translocation into the nucleus. A representative picture from three experiments done independently, which gave the same results, is reported. **(B)** The transcriptional activity of the ERβ protein in BLM cells was analyzed using the pVERE-tk-LUC plasmid (cotransfected with pCMVβ). The results were normalized for β-galactosidase activity. Treatment of the cells with either DPN or E_2_ (10^−8^ M, for 24 h) significantly increased ERβ transcriptional activity. Each experimental group consisted of three replicates and each experiment was repeated three times. Data represent mean values ± SEM. *P<0.05. C, controls.

Experiments were performed to verify whether, in BLM melanoma cells, ERβ might function according to the classical model of estrogen action [11]. By immunofluorescence analysis, we could show that, in BLM cells, most of the ERβ staining was confined in the cytoplasm (Fig 3A); treatment of melanoma cells (24 h) with both DPN and E_2_ (10^−8^ M) induced its nuclear translocation (Fig 3A). Then, we analyzed the effects of ERβ ligands on the transcriptional activity of the receptor in melanoma cells. Fig 3B shows that treatment of BLM cells with either DPN or E_2_ for 24 h significantly increased the transcriptional activation of the ERE-Luc reporter plasmid (normalized for β-galactosidase) indicating that, in BLM melanoma cells, ERβ is associated with the classical transcriptional activity at the nuclear level.

### ERβ agonists affect the expression of cell cycle-related proteins in BLM cells

**Fig 4:**
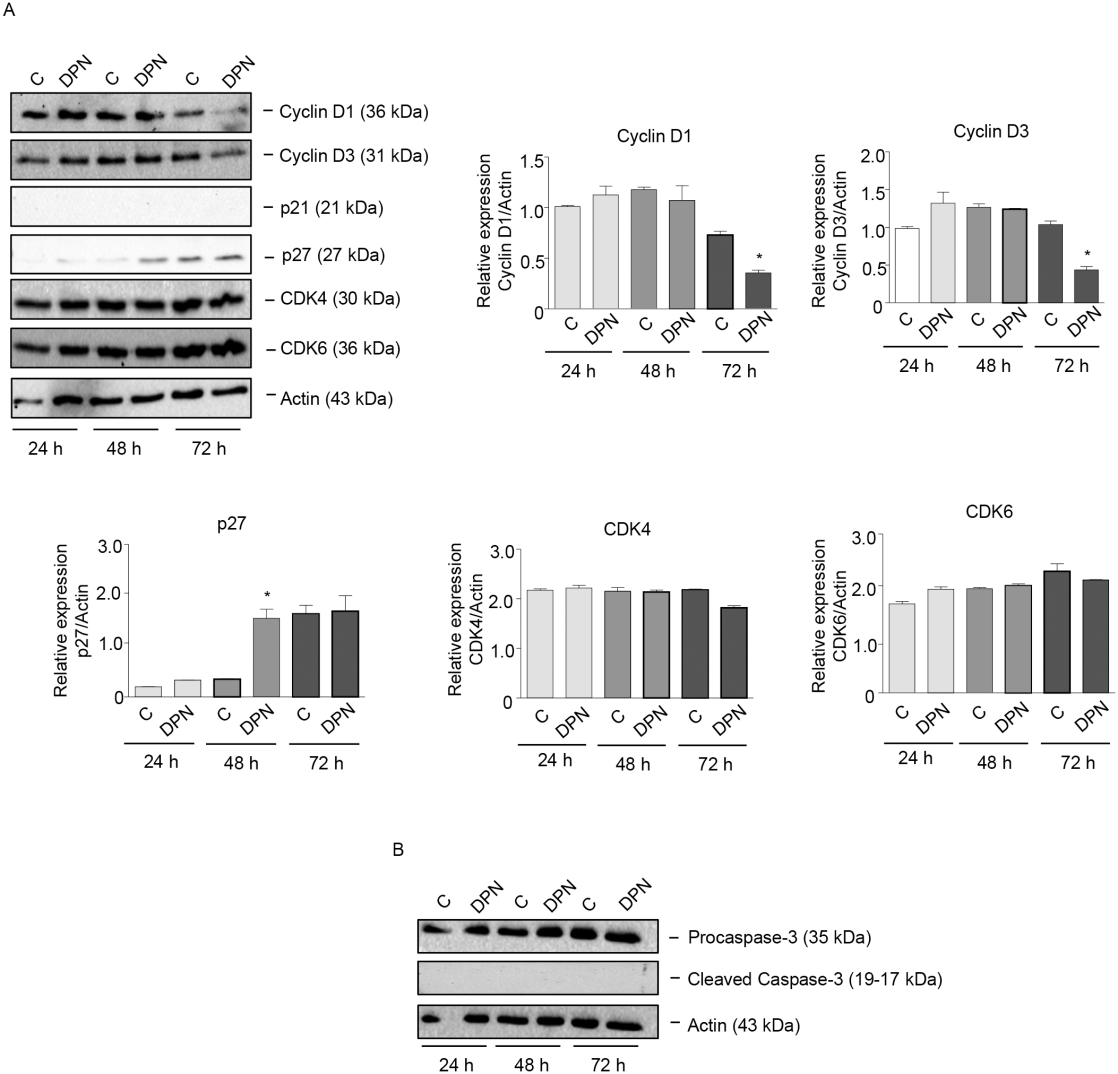
The specific ERβ ligand DPN affects the expression of cell cycle-related proteins in BLM melanoma cells. BLM cells were treated with DPN (10^−8^ M) for 24, 48, or 72 h. Western blot analysis was performed on whole cell extracts by using specific antibodies against cell cycle-related proteins, such as cyclin D1, cyclin D3, p21, p27, CDK4, CDK6 **(A)**, procaspase-3 and cleaved caspase-3 **(B)**. Actin expression was evaluated as a loading control. The treatment with DPN reduced the expression of cyclin D1 and cyclin D3 and increased that of p27, while the levels of cleaved (active) caspase-3 were not affected by the treatment. One representative of three different experiments, which gave similar results, is shown. A statistical evaluation has been performed on the densitometric analysis of the results obtained from the three Western blot experiments performed on cell cycle-related proteins **(A)**.

Estrogens have been shown to affect cancer cell growth through the regulation of proteins involved in cell cycle progression [52]. Experiments were performed to investigate whether ERβ agonists might affect melanoma cell proliferation through alteration of the expression of cell cycle-related proteins. BLM cells were treated with the ERβ agonist DPN (10^−8^ M) for different time intervals (24–72 h). By Western blot assay, we could demonstrate that treatment with DPN induced a significant reduction in the expression of G1 cyclins, such as cyclin D1 and D3 (at 72 h of treatment), and a significant increase in the expression of the CDK inhibitor p27 (at 48 h of treatment) (Fig 4A). On the other hand, the expression of the cyclin D partners CDK4 and CDK6 was not modified by the treatment; the CDK inhibitor p21 was found to be expressed at almost undetectable levels in BLM cells and its expression was not affected by DPN treatment (Fig 4A). Interestingly, DPN did not modify the expression of procaspase-3 as well as that of the cleaved (active) form of caspase-3 (Fig 4B). Taken together, these results indicate that ERβ activation in melanoma cells decreases cell proliferation, through the modulation of the expression of proteins involved in the G1-S progression of the cell cycle, and that the apoptosis pathway is not involved in this activity.

### ERβ activation induces global DNA methylation reprogramming in BLM cells

**Fig 5:**
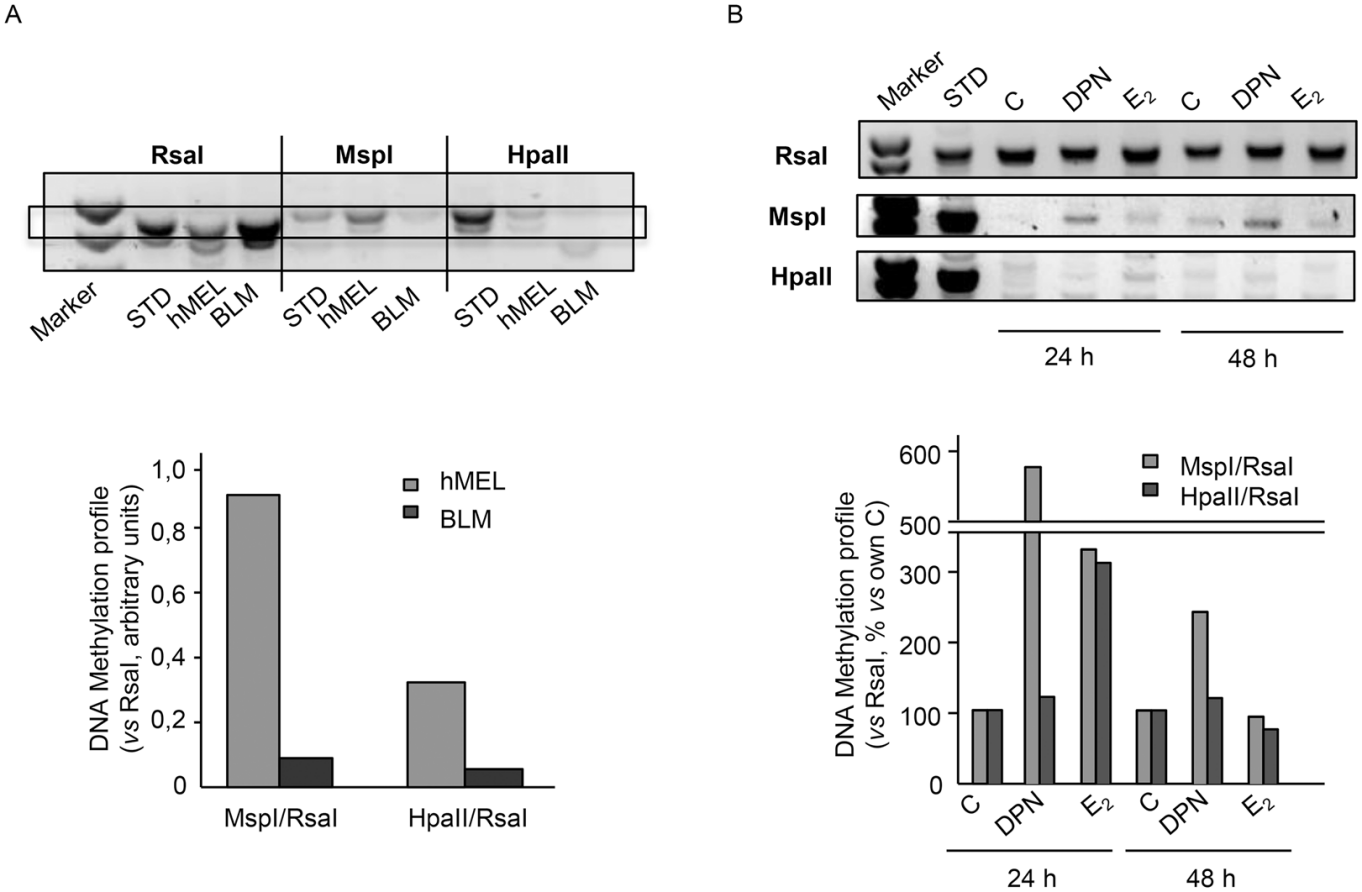
ERβ activation induces global DNA methylation reprogramming in BLM melanoma cells. **(A)** Preliminary experiments were carried out to analyze the global DNA methylation status of BLM cells when compared to that of human normal melanocytes (hMel). To this purpose, a restriction enzymatic assay was employed. For each DNA sample, two restriction digests were performed: one with RsaI and MspI, and one with RsaI and HpaII. RsaI is methylation insensitive, while MspI and HpaII are sensitive to DNA methylation and are able to cut only unmethylated restriction sites. The digests were then amplified by PCR. Data are expressed as the MspI/RsaI or HpaII/RsaI ratios relative to the intensity of the bands. BLM melanoma cells were found to be globally hypomethylated when compared to normal melanocytes, when both MspI and HpaII restriction enzymes were utilized. One representative of three different experiments, which gave similar results, is reported. **(B)** Experiments were performed to evaluate whether activation of ERβ might affect the global DNA hypomethylation status observed in melanoma cells. BLM cells were treated with either DPN or E_2_ (10^−8^M) for 24 or 48 h; the DNA methylation status was then evaluated as described above. Both DPN (at 24 and 48 h) and E_2_ (at 24 h) increased the DNA methylation profile of BLM cells, indicating that ERβ activation reverts the DNA hypomethylation status in melanoma cells. One representative of three different experiments, which gave similar results, is reported.

Experiments were first carried out to analyze the global DNA methylation status of human BLM melanoma cells when compared to that of human melanocytes. To this purpose, a restriction enzymatic assay, utilizing the two methylation sensitive restriction enzymes MspI and HpaII, was performed. These enzymes recognize the same tetranucleotide sequence (5'-CCGG-3') but display different sensitivity to DNA methylation. In particular, MspI does not cut when the external cytosine is methylated while HpaII does not cut when any of the two cytosines is methylated [39,40]. Fig 5A shows that BLM cells are globally hypomethylated when compared to human melanocytes (hMEL), when both MspI and HpaII restriction enzymes are utilized. These data confirm that melanoma cells are characterized by an aberrant global DNA hypomethylation, which is known to be associated with genome instability.

We then evaluated whether activation of ERβ might affect the DNA methylation status of melanoma cells. BLM cells were treated with either DPN or E_2_ (10^−8^ M) for 24 or 48 h; the DNA methylation status was analyzed as described above. Fig 5B shows that DPN significantly increased DNA methylation at 24 and 48 h of treatment, when the MspI restriction enzyme was utilized. On the other hand, E_2_ significantly increased the methylation degree of CG-rich regions at 24 h of treatment, when both restriction enzymes were utilized. These data indicate that ERβ activation reverts the DNA hypomethylation status in melanoma cells and suggest that different ERβ ligands might increase the methylation of the different cytosines of the CG-rich regions (internal *vs*. external) in a specific way.

### ERβ agonists differentially affect the proliferation of melanoma cell lines

**Fig 6:**
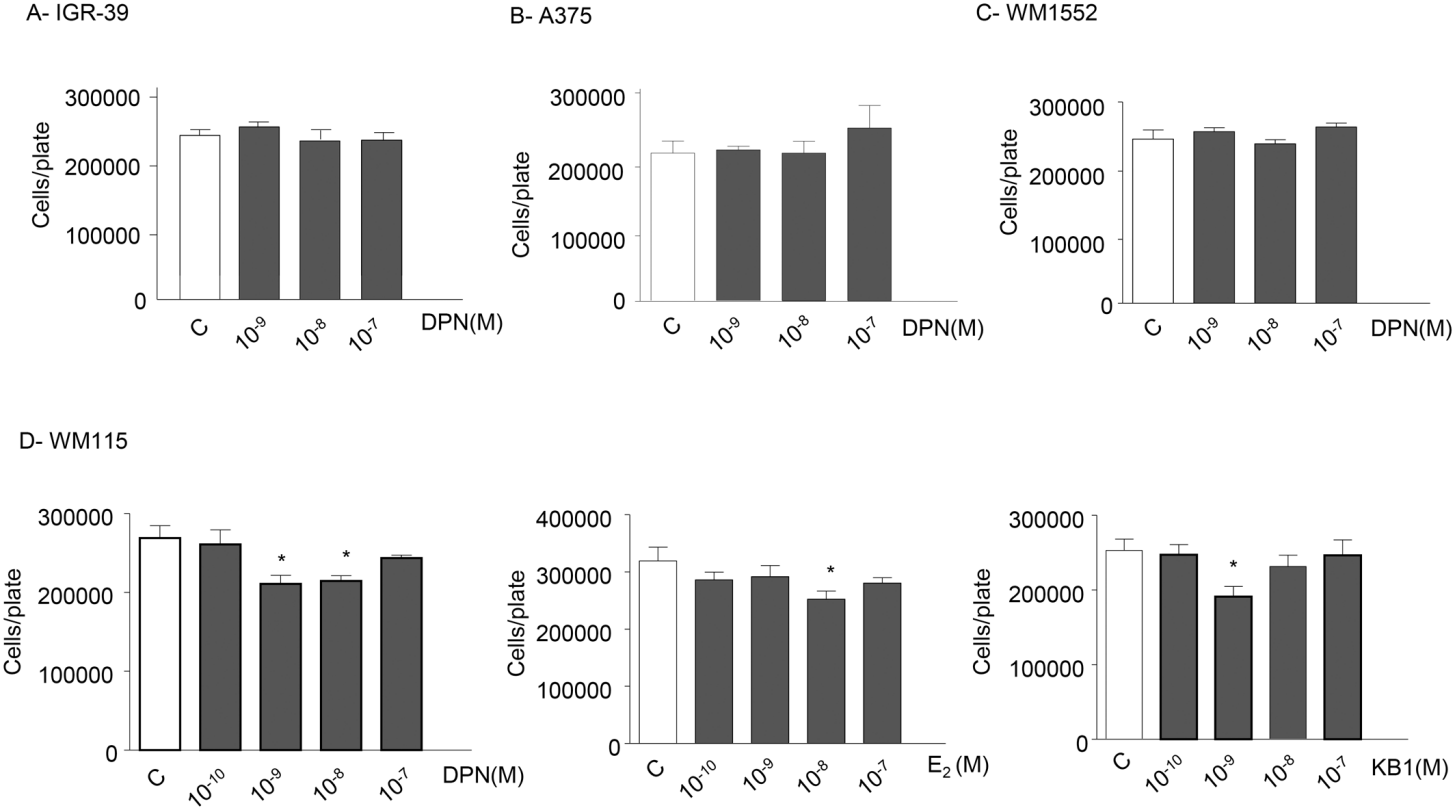
ERβ agonists differentially affect the proliferation of melanoma cell lines. **(A)** IGR-39, A375 and WM1552 melanoma cells were treated with DPN (10^−9^–10^−7^ M) every 48 h for three times. No effect on cell proliferation could be observed in any cell line tested. **(B)** WM115 cells were treated with DPN, E_2_, or KB1 (10^−10^–10^−7^ M) every 48 h for three times. DPN was significantly effective in decreasing cell proliferation at the doses of 10^−9^ and 10^−8^ M. On the other hand, both E_2_ and KB1 significantly reduced cell proliferation at the dose of 10^−8^ M. Each experimental group consisted of six replicates and each experiment was repeated three times. Results are given as cell number/plate. Data represent mean values ± SEM. *P<0.05. C, controls.

Based on the results obtained in BLM melanoma cells (expressing the ERβ receptor subtype and harboring the NRAS mutation), further experiments were performed to assess the effects of ERβ activation on the proliferation of different melanoma cell lines, either lacking the expression of ERβ or expressing ERβ while harboring different oncogenic mutations (*e*.*g*., BRAF). Specifically, the effects of ERβ agonists were assessed on the proliferation of the following human melanoma cell lines: IGR-39 cells (expressing almost undetectable levels of ERβ), A375 and WM1552 cells (expressing ERβ and harboring the BRAF V600E mutation), and WM115 cells (expressing ERβ and harboring the rare BRAF V600D mutation). IGR-39, A375 and WM1552 cells were treated with DPN (10^−9^–10^−7^ M) while WM115 cells were treated with DPN, E_2_ and KB1 (10^−10^–10^−7^ M), as described for BLM cells. As expected, we found that DPN does not affect the proliferation of IGR-39 cells, lacking ERβ expression (Fig 6A). Unexpectedly, and interestingly, the ERβ agonist also failed to affect the growth of A375 and WM1552 melanoma cells, expressing the receptor subtype (Fig 6B and 6C). On the other hand, the proliferation of WM115 cells was reduced by the treatment with DPN, E_2_ and KB1, with a dose-response curve similar to that observed in BLM cells (Fig 6D). Taken together, these results indicate that ERβ activation differentially affects the proliferation of melanoma cell lines. The reasons for these observations are still unclear; however, we might speculate that the efficacy of ERβ agonists in reducing melanoma growth might depend not only on the presence of the receptor but also on other particular features of each melanoma, such as the oncogenic mutation status (NRAS, BRAF) of the tumor.

### Expression of ERβ isoforms in melanoma cell lines

So far, five alternatively spliced transcript variants of the ERβ gene have been described (ERβ1–5) in humans [53]. ERβ wild type, also referred to as ERβ1, is the main variant and ERβ2 and ERβ5 are the most studied splice variants [22]. The expression of these variants has been shown to be tissue-specific and to differentially modulate E_2_ signaling [54].

As discussed above, the reasons for the differential effects of ERβ agonists on the proliferation of melanoma cell lines are still unclear. In addition to the proposed correlation with specific oncogenic mutations, these effects might also be related to the differential expression of ERβ isoforms in the various melanoma cell lines. Based on these observations, by quantitative RT-PCR we analyzed the expression of ERβ1, ERβ2, and ERβ5 in BLM, A375 and WM115 melanoma cell lines.

**Fig 7:**
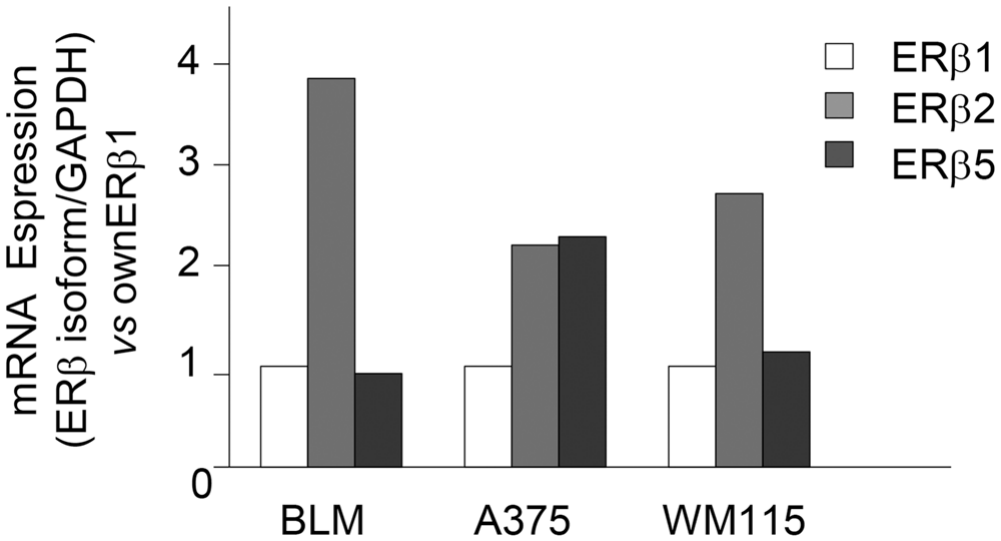
ERβ isoforms (1, 2, and 5) are differentially expressed in melanoma cell lines. The relative expression of ERβ1, 2, and 5 isoforms was evaluated by quantitative RT-PCR, utilizing specific sets of primers. BLM and WM115 cells showed a similar expression of ERβ1 and 5, while expressing higher levels of ERβ2. On the other hand, a high expression of both ERβ2 and 5 isoforms (when compared to ERβ 1) was observed in A375 cells. One representative of three different experiments, which gave similar results, is shown.

Fig 7 shows that the pattern of expression of the ERβ isoforms is similar in BLM and in WM115 cells, with ERβ1and ERβ5 being expressed at similar levels and ERβ2 showing a higher level of expression. On the other hand, in A375 cells, both ERβ2 and 5 are expressed at higher levels than the ERβ1 isoform.

## Discussion

Increasing evidence strongly suggests that ERβ plays a fundamental role in the development and progression of melanoma. In particular, the expression of ERβ was shown to inversely correlate with melanoma progression, being significantly lower in thick melanoma compared with thin melanoma tissues [23,26,27,55]. These observations point toward a potential role of ERβ as a protein associated with suppressive function in this tumor.

In this study, we first investigated the expression of ERβ in a panel of melanoma cell lines; we demonstrated that this estrogen receptor subtype (but not the ERα subtype) is expressed in most of these cell lines.

Then, we analyzed the effects and the possible mechanisms of action of ERβ activation in BLM cells. We could demonstrate that activation of ERβ, achieved by treating the cells with E_2_ or ERβ subtype-selective agonists (the classical ERβ agonist, DPN, or more recently synthesized agonists, KB1, KB2, and KB4) significantly decreased BLM melanoma cell proliferation. This tumor cell inhibitory activity was found to be target protein specific since it was completely abrogated by cotreatment of the cells with the ER antagonist ICI 182,780.

In these experiments, the ERβ agonists displayed bell-shaped responses with growth inhibition at low doses and opposite effect at high doses, as previously reported for cholangiocarcinoma and mesothelioma cells [14,20]. As underlined by Pinton and coworkers [14], this kind of response in not unusual for hormones. The term 'hormesis' has been widely used to describe a biphasic dose response phenomenon characterized by a low dose stressful stimulation and a high dose adaptive response that increases the resistance of the cell to evoked stress [56,57]. A possible explanation could be that cells may increase the production of cytoprotective and restorative proteins which can mediate their adaptive response to the stress induced by ERβ agonists.

In BLM cells, activation of ERβ induced its translocation from the cytoplasm into the nucleus and triggered its transcriptional activity. These data demonstrate that, in these melanoma cells expressing ERβ, this receptor subtype exerts its repressive activity through the classical genomic action of steroid receptors at the nuclear level.

In this paper, we could also show that, in BLM melanoma cells, ERβ agonists exert their antiproliferative activity through the modulation of cell cycle progressing factors (cyclin D1, cyclin D3, p27), without triggering the apoptosis pathway. These data suggest that ERβ activation may inhibit melanoma growth by blocking the G1-S transition phase. Specifically, we could observe that the ERβ agonist DPN significantly reduces cyclin D1 and cyclin D3 protein expression at 72 h of treatment, while increasing the expression of p27 at 48 h of treatment. Since ERβ nuclear translocation and transcriptional activity occur 24 h after treatment of the cells with DPN, we hypothesize that these cell cycle-related proteins might not be directly regulated by ERβ but, more likely, they might be the target of the activity of other direct ERβ downstream proteins. For instance, Wu and coworkers [58] have recently reported that, in bladder cancer cells, the ERβ ligand resveratrol inhibits cell growth through decreased phosphorylation, nuclear translocation and transcription of STAT3, resulting in the downregulation of the expression of STAT3 downstream genes (cyclin D1, survivin, c-Myc and VEGF). Moreover, Nakamura and coworkers demonstrated that, in prostate cancer cells, activation of ERβ induces cyclin D1 expression through increased expression of FOS and JUN; however, according to the data reported, the authors conclude that the interaction of ERβ with the two transcription factors is not direct and likely involves early responsive genes which still need to be identified [59].

Taken together, our results obtained in BLM cells agree with the concept that the antitumor effect of ERβ is associated with altered expression of proteins involved in the cell cycle progression [10,11]. In agreement with the data here reported, ERβ agonists, as well as phytoestrogens (such as apigenin, resveratrol) have been shown to arrest breast cancer cell growth by causing a cell cycle arrest, through the regulation of cell cycle-related proteins, such as cyclin D1 and the CDK inhibitors p21 and p27 [60–62]; more recently, it has been reported that preferential ERβ ligands reduce the expression of the antiapoptotic protein Bcl-2 to increase autophagy in hormone-resistant breast cancer cells [63]. In prostate cancer cells, ERβ agonists inhibit the proliferation rate and the invasive behavior [64,65]. Moreover, ERβ agonists impede prostate cancer epithelial-to-mesenchymal transition, by repressing VEGF-A expression [66]. ERβ ligands were also shown to exert suppressive effects, through modulation of the expression of cell cycle progression proteins, on the growth of tumor cells classically unrelated to the reproductive system, such as colon [18], malignant pleural mesothelioma [14,19,36], lymphoma [21], glioma [67], and cholangiocarcinoma [20] cells. More recently, ERβ agonists have been reported to prevent the development of UVB-induced nonmelanoma skin cancer in mice [68].

It is now well accepted that epigenetic mechanisms play a central role in tumor development. In particular, melanoma cells have been reported to present global DNA hypomethylation, contributing to the genomic instability of tumor cells, when compared to normal cells [28,30]. Thus, reversibility of these epigenetic modifications might represent an effective strategy of treatment for this aggressive form of cancer. In this study, we first confirmed that DNA is globally hypomethylated in human BLM melanoma cells when compared to normal human melanocytes. Then, we could show that treatment of BLM cells with both DPN and E_2_ significantly increased global DNA methylation.

Taken together, our data demonstrate that, in BLM melanoma cells, ERβ activation reduces cell growth, through the modulation of cell cycle related proteins, and that this antitumor activity is associated with the reversal of the global DNA hypomethylation status of these cancer cells.

In this paper, as expected, we could show that ERβ agonists did not affect the proliferation of melanoma cells expressing almost undetectable levels of ERβ (IGR-39).

On the other hand, suprisingly, we found that ERβ agonists were also ineffective in reducing the proliferation of A375 and WM1552 melanoma cells, shown to express the estrogen receptor isoform. At present, the reason for these unexpected results is unclear. However, a possible explanation is that ERβ agonists differentially affect the proliferation of various cell lines, expressing ERβ, according to the cell line-specific oncogenic mutation status. Actually, NRAS and BRAF mutations are very frequently found in melanoma tumors; in particular, BLM cells are NRAS-mutant (a mutation present in about 30% of patients), while both A375 and WM1552 cells harbor the BRAF V600E mutation (the predominant BRAF mutation, occurring in about 50% of cases) [31,69,70]. In melanoma cells, NRAS mutations have been shown to be associated with increased activation of two main downstream signaling pathways: the PI3K/Akt and the MEK/ERK cascades [70,71]. On the other hand, in melanoma cells harboring BRAF mutations, only the MEK/ERK pathway results to be overactivated. Interestingly, ERβ agonists have been shown to exert their significant antitumor/proapoptotic effect through RAS inactivation and specific inhibition of its downstream PI3K/Akt pathway in different cancer cells [11,72,73]. Wang and coworkers [74] have recently reported that ERβ expression inversely correlate with PTEN/PI3K/Akt pathway in triple-negative breast cancer. Moreover, in breast cancer cells, calycosin-induced ERβ activation was associated with a decreased activity of the PI3K/Akt pathway, while the ERK1/2 cascade was not affected by the natural compound [72]. Based on our results as well as on these recently reported observations, we hypothesize that ERβ agonists might effectively reduce the proliferation of melanoma cells harboring the NRAS mutation, through the specific inhibition of the activity of one of the two downstream signaling pathways: the PI3K/Akt cascade. On the other hand, ERβ agonists will not reduce the growth of melanoma cells harboring the BRAF (V600E) mutation, which is associated with the overactivation of the MEK/ERK signaling pathway. Studies are ongoing in our laboratory to confirm this hypothesis.

Taken together, these data would suggest that, in melanoma patients harboring the NRAS mutation, ERβ might represent a novel molecular target for personalized therapeutic strategies, based on ERβ agonists, either alone or in combination with a specific inhibitor of the MEK pathway (*i*.*e*., trametinib). Moreover, these results support the notion that not only the expression of ERβ, but also the genetic analysis of the concurrent oncogenic mutations should be considered to predict the possible response of melanomas to ERβ targeted therapeutic approaches.

In this paper we could also show that ERβ agonists are able to decrease the proliferation of WM115 melanoma cells harboring the BRAF V600D mutation. However, no hypothesis can be suggested in this case, since this is considered a very rare BRAF mutation and very little is known about its associated intracellular signaling alterations; it has actually been reported that, in melanoma cells, BRAF mutations can be associated to different intracellular pathways, in addition to the MEK/ERK cascade [75]. Moreover, whether BRAF inhibitors might have the same effectiveness in patients with this rare BRAF mutation still has to be evaluated [76].

The differential effect of ERβ agonists on the proliferation of the various melanoma cell lines here reported might also be associated with the relative expression of the ERβ isoforms in each cell line. We found that BLM and WM115 cells show a similar pattern of expression of the isoforms with similar levels of ERβ1 and ERβ5, but higher expression of ERβ2. On the other hand, in A375 cells both ERβ2 and ERβ5 are expressed at higher levels than ERβ1. The possible correlation between the expression of the ERβ isoforms and the differential effects of ERβ agonists on melanoma cells is at present unclear. ERβ isoforms have been shown to be co-expressed in various types of tumors, (including breast, ovarian, endometrial, prostate, colon and lung cancers); however, conflicting results have been so far reported on the potential collective effect of their co-existence [41,43,77–81]. In agreement with Hapangama and coworkers [82], we believe that the lack of commercially available specific antibodies for the different receptor isoforms represents a major obstacle in the investigation and clarification of their functions. Based on this observation, most of the functional data so far reported in the literature are only related to ERβ (ERβ1), with little reference to the other alternatively spliced variants.

In conclusion, the data presented in this paper demonstrate that ERβ subtype is expressed in a panel of human melanoma cell lines (BLM, WM115, A375, WM1552). In BLM cells, as well as in WM115 cells, activation of ERβ is associated with a significant and specific antiproliferative effect. In particular, in BLM cells, this antitumor activity is associated with the modulation of the expression of G1-S cell cycle-related proteins and with the reprogramming of global DNA methylation. On the other hand, ERβ agonists failed to affect the proliferation of A375 and WM1552 cell lines. This differential effect of ERβ agonists on the growth of the different melanoma cell lines might be related either to the specific oncogenic mutational status (NRAS, BRAF) or to the relative expression of receptor isoforms in each cell line.

These data confirm that melanoma is a very heterogeneous tumor and support the concept that genetic profiling is mandatory for the development of novel and effective personalized therapeutic strategies for melanoma patients.
